# Modulation of place cells using targeted stimulation with bidirectional microelectrode arrays enhances spatial learning speed in mice

**DOI:** 10.1016/j.fmre.2026.01.004

**Published:** 2026-01-09

**Authors:** Fan Mo, Yilin Song, Guihua Xiao, Zhaojie Xu, Shiya Lv, Wei Xu, Yaoyao Liu, Juntao Liu, Mixia Wang, Yirong Wu, Qionghai Dai, Xinxia Cai

**Affiliations:** a State Key Laboratory of Transducer Technology, Aerospace Information Research Institute, Chinese Academy of Sciences, Beijing 100190, China; b School of Electronic, Electrical and Communication Engineering, University of Chinese Academy of Sciences, Beijing 100049, China; c Beijing National Research Center for Information Science and Technology, Tsinghua University, Beijing 100084, China

**Keywords:** Place cells, Bidirectional microelectrode array, Spatial learning acceleration, Closed-loop neuromodulation, Cognitive enhancement technology

## Abstract

Spatial cognition plays a critical role in survival and development, yet effective technologies for enhancing this capability remain scarce. This study investigates a neural modulation approach targeting place cells—specialized hippocampal neurons responsible for spatial representation. Bidirectional microelectrode arrays with micron-level spatial precision were developed using micro-electromechanical systems technology, integrating detection, stimulation, and shielding functions. The array enables simultaneous electrophysiological recording and confined electrical stimulation while minimizing signal interference. A modified Y-maze behavioral paradigm and electrical stimulation protocol were designed to assess spatial learning enhancement. Experimental results demonstrated that mice receiving 72 mV/90 Hz electrical stimulation through the array achieved comparable place cell activation patterns and behavioral performance after 1 week of training to control mice requiring 2 weeks of conventional training. Quantitative analysis revealed a 19.7% increase in learning trajectory success rate, 50.2% increase in spatial information, and 25.1% reduction in area of place field in stimulated subjects compared to non-stimulated controls. These findings indicate that precise electrical modulation of place cell ensembles through the developed array can significantly accelerate spatial learning processes. This integrated neural interface technology provides a closed-loop solution for targeted neuromodulation, offering new possibilities for enhancing spatial cognition through direct neural circuit engagement.

## Highlights

• Bidirectional MEMS array enables precise hippocampal recording/stimulation (80 µm).
• A 7-day stimulated learning achieves comparable performance to 14-day natural spatial navigation.
• A 72 mV/90 Hz stimulation boosts spatial information while reducing place field size.
• Closed-loop system enables real-time detection and modulation of place cell activation patterns.
• Framework advances cognitive prosthetics for memory enhancement.

## Graphical abstract

**Figure:**
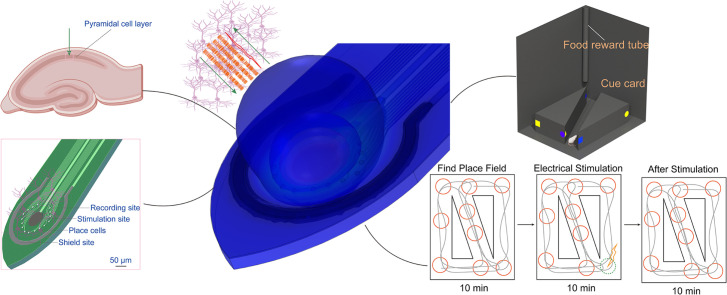

## 1 Introduction

The ability to precisely regulate neurons with specific functions is of great significance for understanding and modulating brain mechanisms. The hippocampus serves as the neural substrate for spatial cognition through its specialized place cell networks that construct cognitive maps of environmental space [1,2]. These spatially-tuned neurons not only mediate navigation and memory formation [3], but exhibit remarkable plasticity during spatial learning—a property that remains underexploited in neuro-engineering applications. A significant body of research has demonstrated that regulating the activity of place cells can significantly influence an animal's spatial behavior and memory abilities [4,5]. However, the random anatomical distribution of place cells within hippocampal CA1/CA3 regions creates fundamental technical barriers [6], as they tend to form small, localized spatial clusters [7]. This spatial heterogeneity poses unique challenges for selective neuromodulation, as traditional stimulation electrodes lack cellular specificity.

To achieve precise regulation of these cells, functional identification through electrophysiological signatures must precede intervention. This closed-loop paradigm requires spatiotemporally aligned recording-stimulation systems—a requirement fundamentally unmet by conventional separated electrode configurations. This provides crucial reference data for subsequent electrical stimulation. In recent years, electrophysiological techniques have made significant progress in recording and analyzing neuronal activity, particularly with the development of multi-electrode array technology [8]. These techniques enable researchers to simultaneously monitor the activity of multiple neurons, providing a more comprehensive understanding of the dynamic properties of neural networks. However, traditional electrode devices still have limitations in terms of precision in detection and stimulation, as well as real-time regulation capabilities. These shortcomings become more pronounced in applications requiring precise regulation of functional cells such as place cells [9,10]. To achieve precise regulation of neurons with specific functions, in addition to relying on high-precision electrophysiological detection devices, a highly compatible backend circuit system is also required [11,12]. Although existing circuit designs can meet these requirements to some extent, signal distortion is often introduced during the signal processing and transmission process, which can, in turn, affect the stimulation effectiveness [13]. Therefore, the development of integrated detection-stimulation electrode systems is critical to achieving precise neuronal regulation.

When regulating place cells, the electric field spreads outward along low-impedance pathways, leading to unintended activation or inhibition of neurons in non-target areas. With the rapid advancement of micro-nano fabrication technologies, delivering smaller stimulus currents through microelectrodes can enable targeted stimulation, thereby modulating neuronal activity. Microstimulation allows clinicians and researchers to selectively control neuronal firing, encode information for direct communication with the nervous system, and achieve precise regulation of specific brain regions or populations of neurons [14]. Using high-density microelectrode arrays, neurons can be selectively excited or inhibited at the single-cell level, successfully revealing neural network activity patterns [15]. By designing ground sites around the stimulation site, the stimulation area can be further restricted [16]. This selective directional stimulation significantly enhances the effectiveness of electrical stimulation while minimizing side effects [17]. Although microelectrodes partially limit the spread of stimulation effects, they still fail to achieve precise regulation of place cells. Optimizing stimulation patterns can enable precise control of neuronal activity. Implantable neural electrical stimulation relies on biphasic charge-balanced pulses interacting with the nervous system, where parameters such as frequency, intensity, and pulse width directly determine neuronal excitability and network responses [18]. By adjusting waveform shape, symmetry, and duration, fixed stimulation patterns can be designed to more precisely control the activation of neurons, achieving more accurate regulatory effects [19].

After the electrode array is implanted in the brain, a double capacitance effect occurs between the electrodes and the cerebrospinal fluid [20]. This effect can make the stimulation signals received by neurons uncontrollable, thereby affecting the effectiveness [21]. Establishing a double capacitance model requires comprehensive consideration of the electrode material properties, geometric structure, and interactions with the surrounding biological environment [22,23]. Using highly conductive materials can effectively reduce the resistance between the electrodes and the tissue, thereby minimizing the impact of the double capacitance effect [24]. Nevertheless, the double capacitance effect remains unavoidable, making it particularly important to establish an accurate equivalent circuit model for the double capacitance [25,26]. Through in-depth research on the double capacitance effect, we can better understand the potential issues in signal transmission under different experimental conditions, thereby optimizing the settings for electrical stimulation parameters.

This study presents an integrated neural interface system comprising a bidirectional electrode array (Targeted Stimulation Bidirectional Microelectrode Array, TS-BMA) and its companion signal processing circuitry. Leveraging precision MEMS fabrication techniques, we engineered a neuroelectronic interface capable of simultaneous high-resolution neural recording and spatially constrained electrical stimulation. The TS-BMA architecture achieves localized field confinement through optimized electrode geometry and current steering protocols, enabling targeted modulation of hippocampal place cell populations. We then implemented a modified Y-maze paradigm to validate system efficacy, which revealed that TS-BMA-mediated burst stimulation (72 mV, 90 Hz) produced significant neuroplasticity enhancement. Stimulated groups exhibited comparable place cell activation patterns and maze navigation performance after 7-day training to control groups requiring 14-day training. This closed-loop neuromodulation platform establishes a method for accelerated spatial learning through direct neural stimulation, with potential applications in neuro-prosthetics and cognitive enhancement technologies.

## 2 Materials and methods/experiment

### 2.1 Animals

All animal experiments were approved by the Beijing Laboratory Animal Care Association and by the Animal Care and Use Committee at the Aerospace Information Research Institute, Chinese Academy of Science (AIRCAS) (Ethics Approval Number: AIRCAS-202302-01). All mice were placed in a 12-h light/dark cycle. All experiments were performed during the dark cycle. Electrophysiology experiments were performed at the Aerospace Information Research Institute. Male (8-week-old) WT mice (C57BL/6N) were obtained from Charles River.

### 2.2 Study design

This study investigates spatial cognition and hippocampal place cell activity in mice using a behavioral maze combined with electrophysiological recording and electrical stimulation techniques. Behaviorally, an improved Y-maze equipped with cue cards of different colors and shapes was used. Mice underwent pre-surgical environmental adaptation (2 weeks), microelectrode implantation surgery (targeting the pyramidal cell layer of the hippocampal CA1 region), and post-surgical recovery (1 week). Training was divided into two phases: (1) Exploration training (2 weeks): food-restricted mice randomly explored the maze for scattered food, developing continuous running ability (>30 min); (2) Path memory training (2 h/mouse): random food was removed, feeders were placed at two corners of the maze, and food was provided only after completing a specific “reward path,” reinforcing path learning. In formal experiments, no food rewards were given, and electrical stimulation experiments were conducted: TS-BMA was used to implant electrodes in the hippocampal CA1 region to record neuronal firing, while electrical stimulation was applied at specific locations, followed by recording changes in place cell activity and behavioral alterations. For place cell analysis, the behavioral space was divided into a 1 cm grid to construct raw firing maps and occupancy maps. After Gaussian kernel smoothing, smoothed firing rate maps (firing map/occupancy map) were generated. Analysis excluded firings when speed was <2 cm/s, and firing locations were superimposed onto the Gaussian distribution of trajectory points.

In addition, more details during surgery and the details of behavior procedures, electrophysiological recording and stimulation, the detailed fabrication process of the microelectrode, histological verification of the microelectrode implantation location, modification of the microelectrode, in vitro electrical stimulation experiment setup, EIS experiment setup and double layer capacitor circuit model, and place cell analysis are presented in the supplementary materials.

## 3 Results and discussion

### 3.1 Design and simulation of targeted stimulation bidirectional microelectrode array

**Fig. 1:**
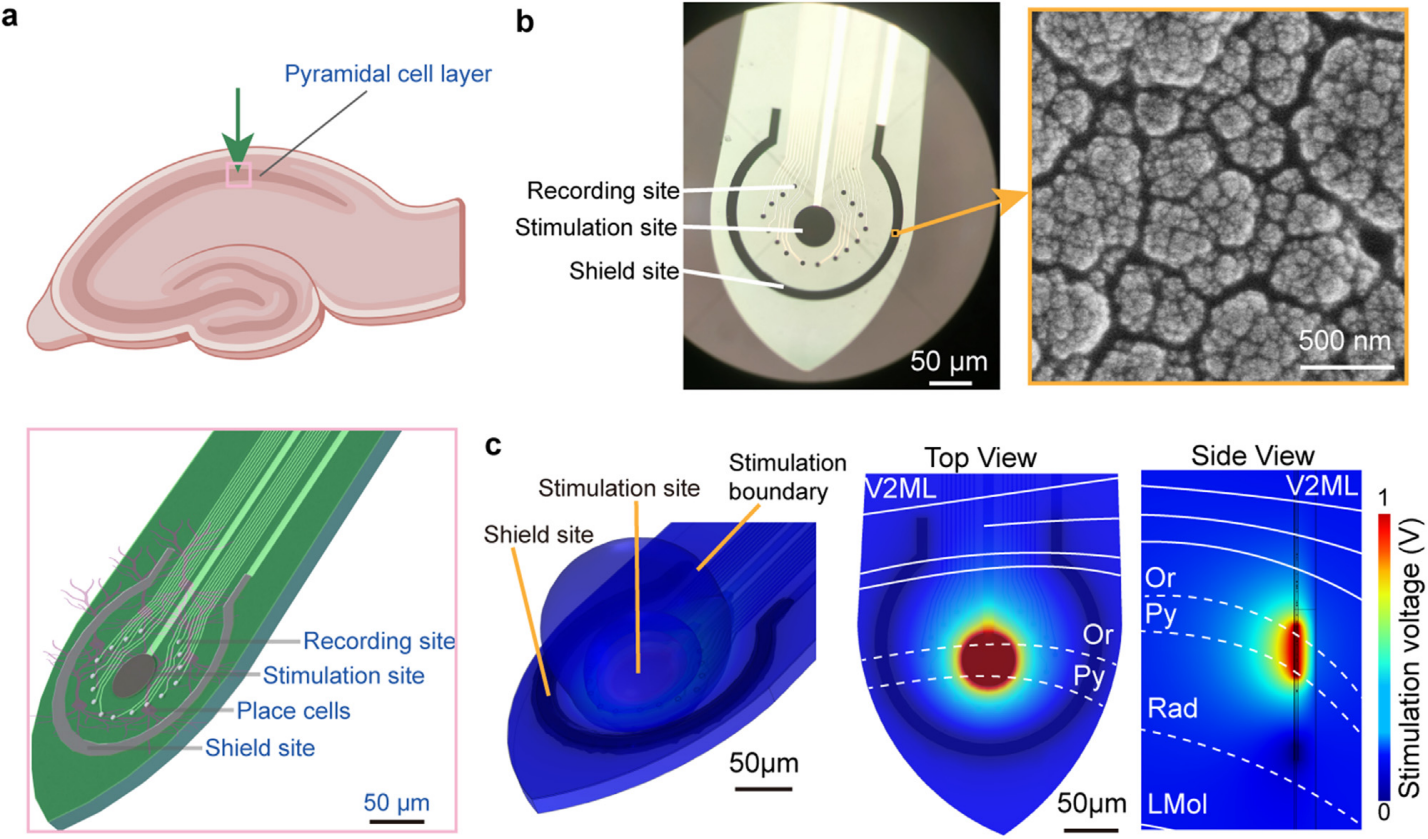
**Schematic of the TS-BMA for recording and limited area stimulation in the dorsal CA1 pyramidal cell layer.** (a) Schematic of the microelectrode implantation and design. (b) TS-BMA and scanning electron microscopy imaging of the site surface. (c) Simulation of the electrical stimulation region. Or, Stratum oriens; Py, Stratum pyramidale; Rad, Stratum radiatum; LMol, Stratum lacunosum-moleculare; V2ML, Ventral blade molecular layer.

The TS-BMA was specifically designed for bidirectional detection and stimulation in the hippocampal CA1 pyramidal cell layer (Fig. 1a), where place cells are densely distributed. The array architecture integrates sixteen 8-µm electrophysiological recording sites surrounding a central stimulation site (50 µm diameter). Fabricated using micro-electromechanical systems (MEMS) technology, the array measures 6 mm in length and 27 µm in thickness (Fig. S1a). The manufacturing process (Fig. S1a) began with a silicon-on-insulator (SOI) substrate, with key technical steps including thermal oxidation, conductive layer patterning, and deep reactive ion etching (DRIE). Post-fabrication, aluminum wire bonding (Fig. S1b) connected electrode pads to a custom 20-channel ceramic package, ensuring reliable electrical connections between the electrode array and backend circuitry. Histological validation using Dil dye tracing (Fig. S1c) confirmed precise implantation in the dorsal CA1 pyramidal layer.

Platinum nanoparticle modification was applied to all electrode surfaces to reduce recording site impedance and enhance stimulation site transmission stability (Fig. 1b). To precisely control stimulation range, the electrode design incorporated a 20-µm-wide open shielding ring structure that effectively confined electrical stimulation to target areas. Finite element simulations (Fig. 1c) demonstrated that this shielding configuration restricted electric field boundaries, concentrating field intensity near the stimulation site with significant local enhancement effects while minimizing off-target neural activation.

A 3D-printed protective enclosure was implemented for circuit system shielding, with additional aluminum foil wrapping to reduce electromagnetic interference (Fig. S1d). The integrated backend circuitry (Fig. S1d, S1f) combined a 16-channel recording module with a programmable stimulator to establish a closed-loop system for synchronous detection and stimulation. The stimulation circuit comprised a gain module (connecting the STG4002 signal generator to ground) and an attenuation module (linking the TS-BMA stimulation site to the shielding ring). The electrophysiological circuit included a voltage reference source with 16 parallel signal amplification and filtering channels, each independently connected to TS-BMA recording sites.

By integrating concentric recording/stimulation sites with micron-scale shielding, the TS-BMA system enables precise local stimulation of target neurons while detecting neuro-electrophysiological signals, addressing a critical limitation in hippocampal neuromodulation: the inability to selectively target functionally distinct yet spatially intermingled neurons. This closed-loop functionality, coupled with spatial selectivity in electrical stimulation, provides a technical foundation for the precise regulation of hippocampal place cells and targeted modulation of place cell networks. This study’s electrical stimulation method identifies place cells through electrophysiological signals, combined with precise local electric field stimulation (without photodamage or physical interference), eliminating the reliance on molecular markers and photoelectric artifacts associated with optogenetic stimulation [27,28]. Its non-genetic modification characteristic avoids immune rejection and ethical risks [29,30], and it is directly compatible with clinical DBS systems, offering advantages in specificity, real-time performance, and potential for clinical translation.

### 3.2 Electrophysiological and stimulation performance characterization

**Fig. 2:**
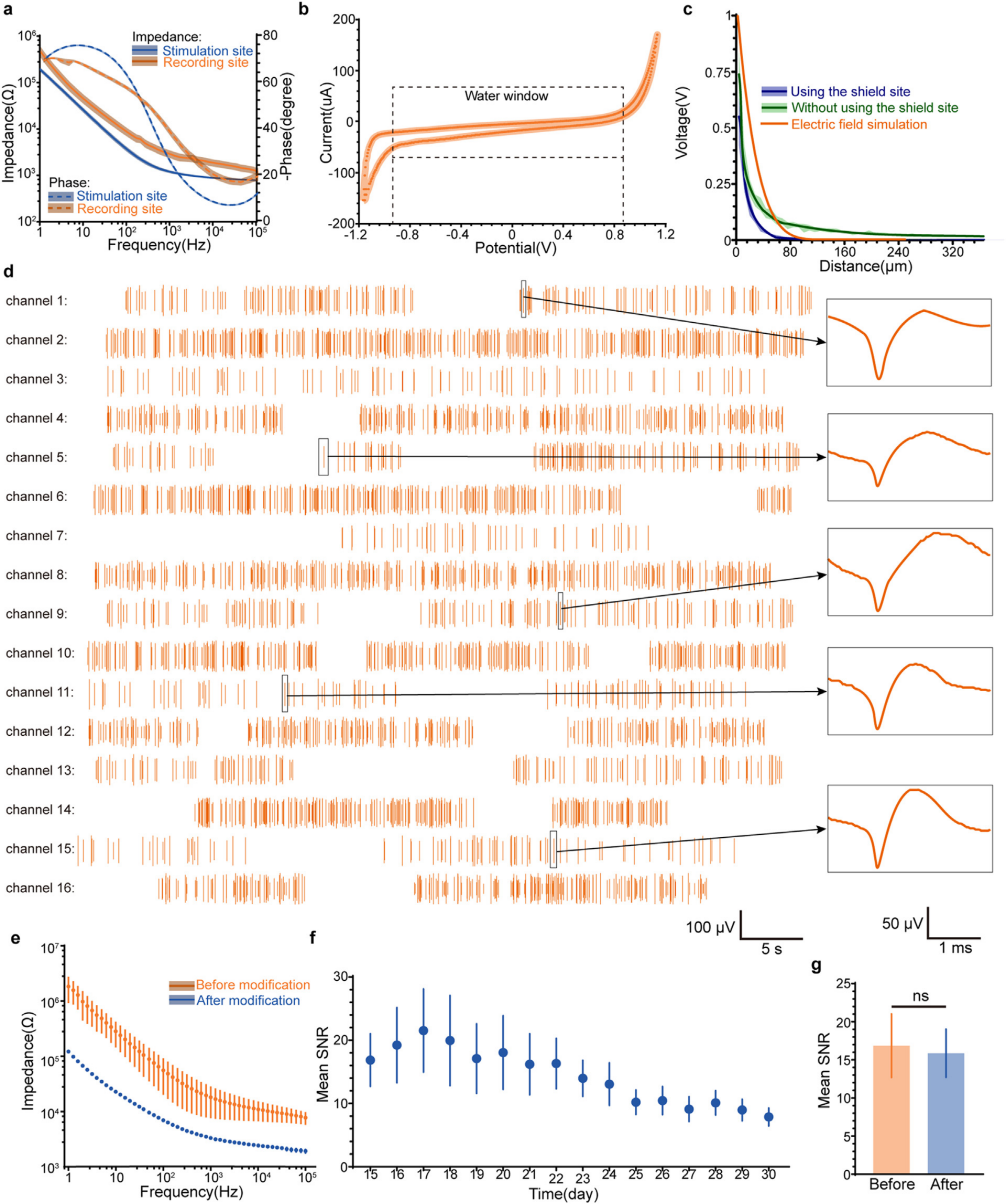
**Electrophysiological detection and electrical stimulation performance of TS-BMA.** (a) Impedance and phase characteristics versus frequency for the electrophysiological sites and the stimulation site of TS-BMA. (b) Cyclic voltammetry measurement of the electrochemical stability window in physiological saline. (c) In vitro experimental and simulation validation of the stimulation electric field. (d) 16-channel spike sequence (left) and partial spike waveform (right). (e) Impedance changes before and after modification. (f) Signal-to-noise ratio (SNR) changes after TS-BMA implantation. (g) Comparison of SNR before and after stimulation (n = 80, *p* = 0.254). Statistical significance was assessed by the Mann-Whitney U test. ns., not significant. *p < 0.05, **p < 0.01; ***p < 0.001, ****p < 0.0001. All error bars show SD.

A stable electrode-brain tissue interface is crucial—any signal distortion could lead to misinterpretation of place cell activity or delivery of incorrect stimulation. The stability of this interface was rigorously validated through electrochemical impedance spectroscopy (EIS). The TS-BMA demonstrated stable electrochemical performance. Platinum nanoparticle-modified recording sites exhibited impedance values of 5–10 kΩ at 1 kHz and maintained flat phase responses across the 1 Hz to 10 kHz range (Fig. 2a). Cyclic voltammetry revealed an electrochemical stability window of approximately 1.8 V for stimulation sites in saline solution (0.9% NaCl, 37 °C), safely accommodating stimulation amplitudes up to 800 mV without water electrolysis (Fig. 2b).

To further validate TS-BMA's localized stimulation performance, we conducted in vitro electric field measurements following finite element simulations (see supplementary materials: Methods 1.7). The in-vitro electric field mapping (Fig. 2c) confirmed spatial confinement of stimulation effects, with the majority of the electric field concentrated within an 80 µm radius spherical region. Experimental measurements show that, with the addition of a 20 µm shielding ring, the effective radius of the electric field is approximately 80 µm, whereas without the shielding ring, the effective radius exceeds 320 µm. Compared to the simulation without a shielding ring, the electric field decays more rapidly when using the shielding ring, with an effective radius of 100 µm observed in the shielded configuration. Simultaneously, the 16-channel recording capability successfully captured place cell spike activity during electrical stimulation (Fig. 2d), verifying TS-BMA's bidirectional functionality. We implanted TS-BMA into the CA1 region of the mouse hippocampus for electrophysiological recording. We found that all 16 channels were able to detect electrophysiological signals, and the spike waveforms were clear and complete, meeting the requirements for detecting place cells.

Furthermore, we systematically evaluated the charge storage capacity (CSC) and charge injection limit (CIL) of the stimulation sites using electrochemical scanning, with CSC = 29.49 ± 7.71 *mC*⋅*cm*^-2^ and CIL = 1.18 ± 0.068 *mC*⋅*cm*^-2^ (see supplementary materials: Methods 1.10).

After modification with platinum nanoparticles, the electrode impedance decreased by approximately a factor of 10 (Fig. 2e). To quantify biocompatibility, we tracked the signal-to-noise ratio (SNR) of 80 recording sites across five implanted mice over 15–30 days. The results showed that the SNR remained stable at 7.88 ± 1.42 after 30 days of implantation (Fig. 2f), directly demonstrating the long-term stability and excellent biocompatibility of the electrode interface, ensuring the sustained acquisition of high-quality electrophysiological signals over weeks. We observed a modest signal-to-noise ratio decline, a common phenomenon in chronically implanted electrodes primarily attributed to glial scar formation, chronic inflammation, and mechanical micromotion [[31], [32], [33]]. Future improvements may include integrating dexamethasone-eluting systems [34] to suppress inflammatory responses or employing flexible conductive hydrogel encapsulation [35] to reduce mechanical mismatch. These strategies, synergizing with platinum nanoparticle modification, could fundamentally enhance long-term recording stability.

By comparing the 16-channel raw signals during the application of 72 mV/90 Hz electrical stimulation (lasting 1 s) with the resting period before stimulation (1 s), the SNR was calculated (Fig. 2g). The data showed that the SNR during stimulation was 15.88 ± 3.14 (n = 80), with no statistically significant difference compared to the baseline SNR before stimulation (16.87 ± 4.14, *p* = 0.254). Stimulation artifacts were effectively suppressed by the shielding ring design.

The constrained stimulation range (< 80 µm) minimizes unintended modulation of non-target neurons, addressing a critical challenge in hippocampal neuromodulation. These performance metrics collectively enable precise closed-loop operation, resolving the spatial mismatch inherent in traditional separated-electrode systems.

### 3.3 Stimulation waveform optimization and signal transmission modeling

Biphasic square wave electrical pulses are the preferred waveform for neuromodulation due to their charge-balanced characteristics, effectively avoiding electrode polarization and tissue damage. Through multiple experiments, this study selected 90 Hz (gamma band) as the core stimulation frequency, given its critical role in regulating place cell activity. The physiological basis for selecting 90 Hz (Gamma band) as the core stimulation frequency stems from the critical role of Gamma oscillations (30–100 Hz) in spatial cognition. Previous studies have confirmed that Gamma oscillation frequencies guide information flow in the hippocampus [36], coordinate synchronized firing of hippocampal place cells, significantly enhance the efficiency of spatial information encoding [37,38], and effectively drive network reorganization and place field reconstruction of place cells [39], thereby supporting the optimization of spatial behavior.

**Fig. 3:**
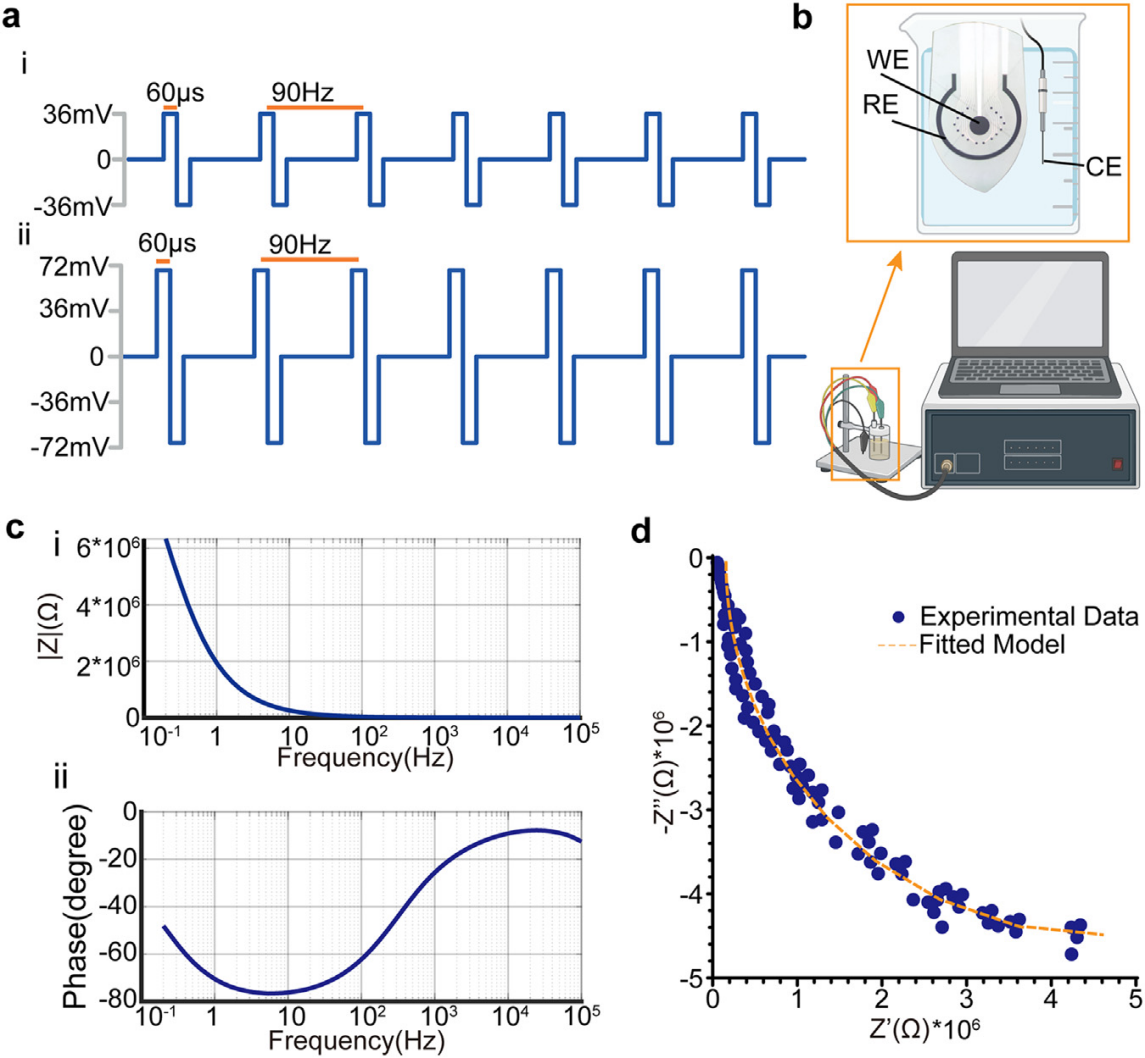
**Design of electrical stimulation waveform and time-frequency analysis of the output waveform.** (a) Biphasic square wave electrical pulse. (i) 36 mV (ii) 72 mV. (b) EIS experimental setup. WE, Working Electrode; RE, Reference Electrode; CE, Counter Electrode. (c) EIS impedance spectrum (i) and phase spectrum (ii). (d) Parameter fitting of the double-layer capacitor circuit model.

To validate the threshold-dependent effects of stimulation intensity on plasticity, the study designed control waveforms with amplitudes of 36 mV (subthreshold) and 72 mV (effective threshold) (Fig. 3a-i, ii).

The double-layer capacitance formed at the electrode-tissue interface leads to distortion of the stimulation signal. To quantify this effect, the study established an equivalent circuit model (Fig. S2a) between the stimulation site and the shielding electrode through electrochemical impedance spectroscopy (EIS) testing (Fig. 3b). The impedance magnitude and phase were systematically measured over the frequency range of 0.1 Hz to 100 kHz (Fig. 3c), and the double-layer capacitance and charge transfer resistance were obtained using linear fitting (Fig. 3d, *R*^2^ = 0.92).

The model indicates that the stimulation signal undergoes frequency-dependent attenuation during transmission: unit impulse response analysis shows that the system has a significant filtering effect on low-frequency components below 10 kHz (Fig. S2b). After the input waveform passes through the transmission model (Fig. S2c), the output signal exhibits slight distortion in the time domain (Fig. S2d) and a loss of low-frequency energy in the frequency domain (Fig. S2e). Notably, the gamma frequency band (30–100 Hz) signals related to place cell activity did not attenuate, suggesting that the model maintains high fidelity within the target frequency band. This characteristic explains the effectiveness of the 90 Hz stimulation in subsequent experiments—despite overall signal attenuation, the key frequency components were preserved, ensuring rhythmic driving of the place cell population.

The combination of biphasic square waves with gamma frequencies essentially transforms biophysical mechanisms into engineering control strategies. The 90 Hz stimulation not only matches the intrinsic oscillatory properties of place cells, but the study, by quantifying the signal distortion mechanism (Fig. S2e), also provides a theoretical basis for subsequent closed-loop compensation algorithms—for example, for attenuation characteristics below 10 kHz, digital pre-emphasis techniques can be used to restore the original waveform fidelity.

### 3.4 Spatial learning dynamics in mice

**Fig. 4:**
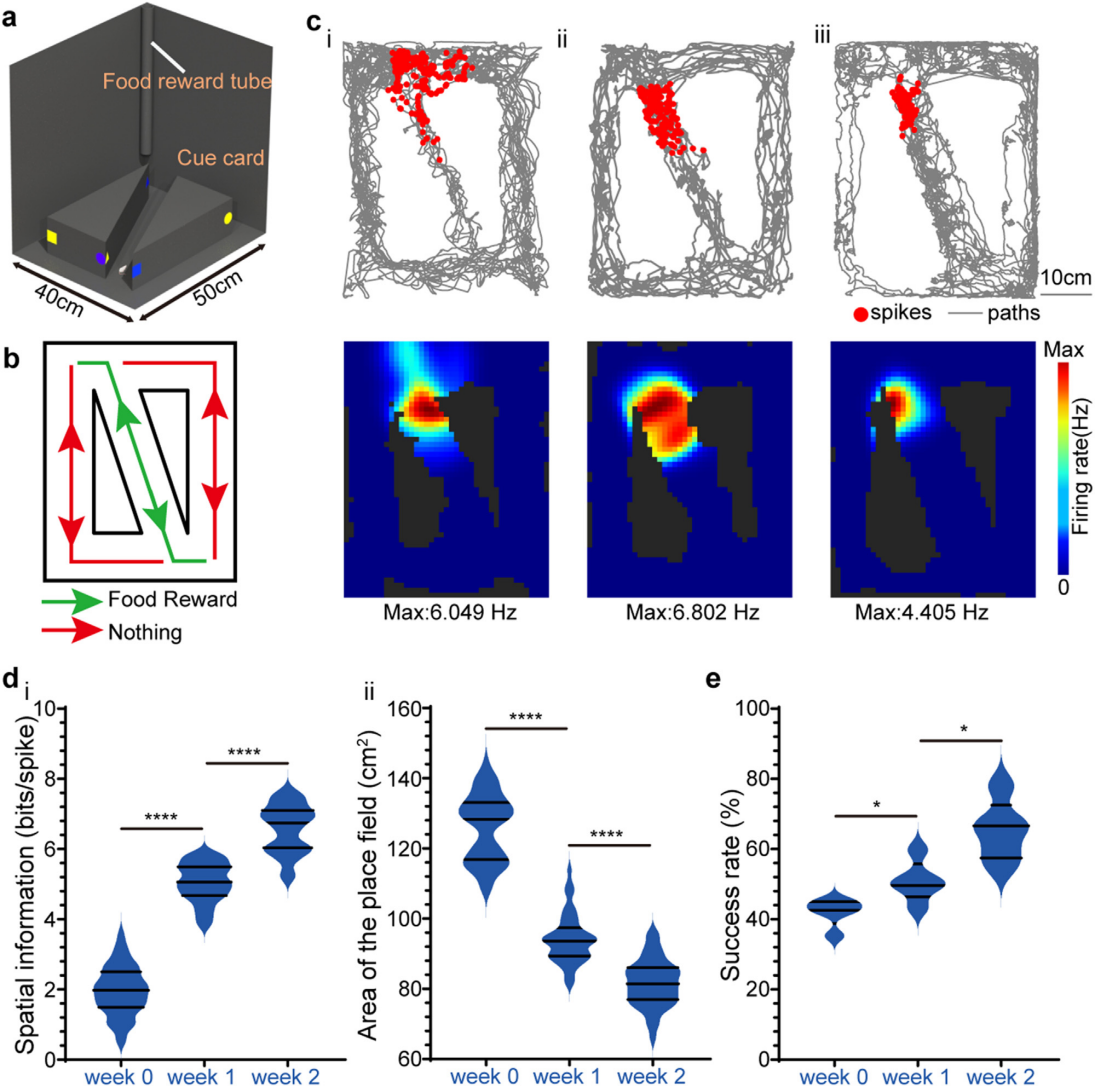
**Spatial learning process in mice.** (a) Experimental maze design. (b) Behavioral experiment design. (c) Three representative examples of paths (gray) with spike locations (red) and place field heat maps of mice after 1 week (i), 2 weeks (ii), and 3 weeks (iii) of spatial learning training. (d) Changes in place cell activity in mice after 1 week (n = 50), 2 weeks (n = 44), and 3 weeks (n = 39) of training. (i) Place field spatial information. (ii) Place field area. (e) Changes in the success rate of mice (n = 5). Statistical significance was assessed by the Mann-Whitney U test. ns., not significant. *p < 0.05, **p < 0.01; ***p < 0.001, ****p < 0.0001. The three horizontal lines in the middle correspond to the first quartile, median, and third quartile in the violin plot.

To analyze the neural execution of spatial learning's neural dynamics and provide baseline data for subsequent studies, the study designed an improved Y-maze behavioral maze (Fig. 4a). The maze has food reward tubes set at the ends of the three distal arms, with the central intersection area designed as the navigation path (width 10 cm). During the training phase, a differential reinforcement strategy was employed: mice triggered food rewards (0.1 mL of 10% sucrose solution) only when passing through the central path, while no rewards were given when traveling along the edge paths (Fig. 4b). In the testing phase, all rewards were removed, and the spontaneous navigation strategies of the mice were quantified using a trajectory tracking system (50 Hz sampling rate).

After 1 week of training, the mice exhibited exploratory navigation patterns (Fig. 4c-i), achieving a 42% average dwelling probability in the maze's central zone (success rate). The spatial firing heatmaps of the corresponding place cells showed diffuse place fields (average area 126 cm²), with an average spatial information content of 1.97 bits/spike (n = 50 cells). By the second week (Fig. 4c-ii), the central path occupancy rate increased to 50.8%, and the average place field area significantly decreased to 94.3 cm² (*p* = 4.19E-32), with the average spatial information content increasing to 5.05 bits/spike (n = 44 cells, *p* = 2.05E-40). After the third week of training (Fig. 4c-iii), the mice exhibited goal-directed navigation (average central path usage rate of 65.3%), with the average place field further sharpening to 81.6 cm² (*p* = 2.33E-12, compared to the second week), and the average spatial information reaching 6.58 bits/spike (n = 39 cells, *p* = 4.32E-17). Neuron counts decreased over time due to gradual electrode displacement; recordings were excluded if the signal-to-noise ratio dropped below 3:1. During spatial learning in mice, at the microscopic level, the spatial information of place cells increases, and the area of place fields decreases; at the macroscopic level, the success rate continuously increases (Fig. 4d).

### 3.5 Enhanced spatial learning through targeted stimulation on place cells

**Fig. 5:**
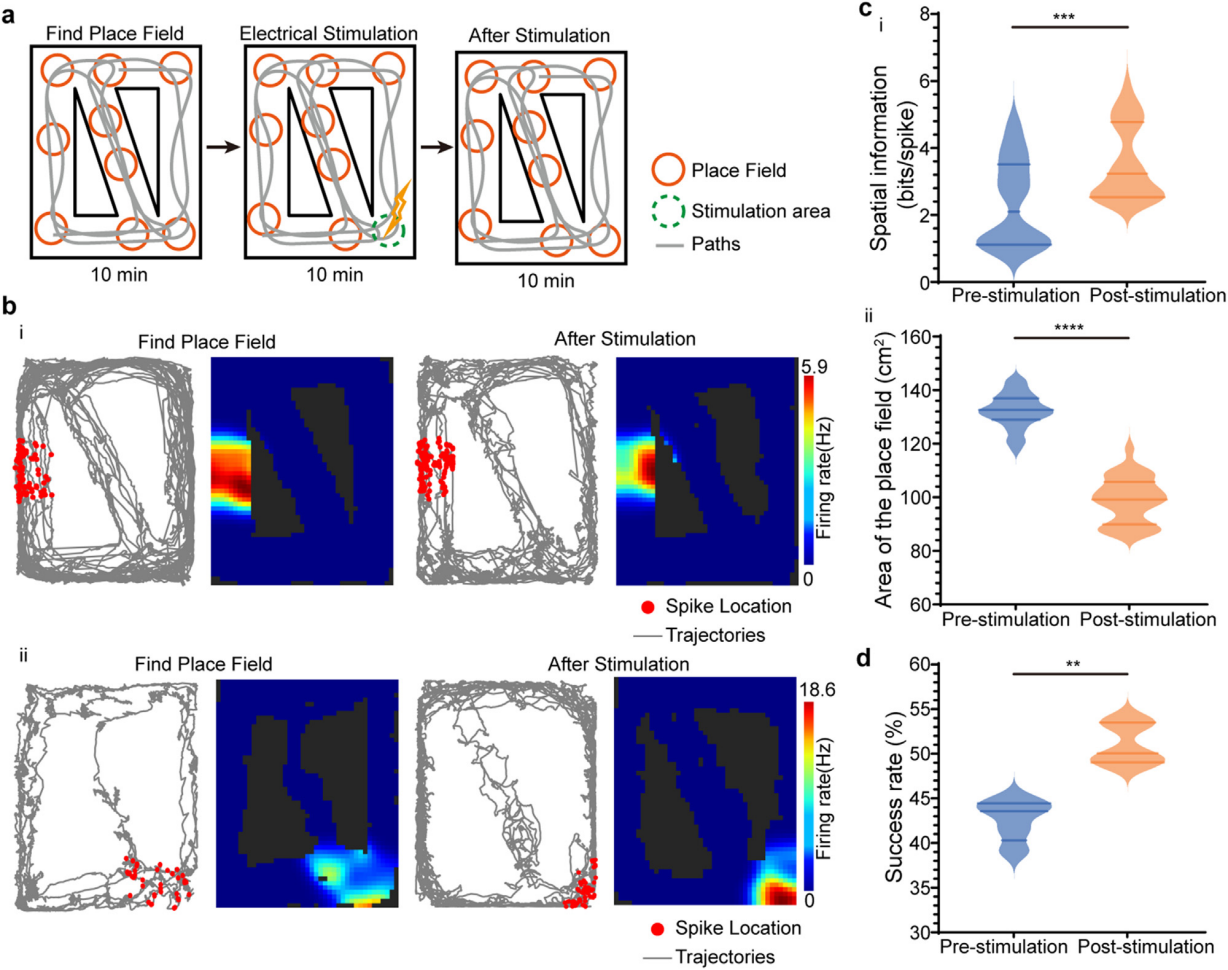
**A 72 mV 90 Hz electrical stimulation enhances spatial learning speed in mice.** (a) Behavioral experimental design of electrical stimulation. (b) Two representative examples of behavioral trajectory changes and place cell activity changes in two groups (i, ii) of mice after 72 mV 90 Hz electrical stimulation. (c) Changes in place cell activity in mice before and after stimulation. (i) Place field spatial information (n = 28). (ii) Place field area (n = 28). (d) Changes in the success rate of mice (n = 5). Statistical significance was assessed by the Mann-Whitney U test. ns., not significant. *p < 0.05, **p < 0.01; ***p < 0.001, ****p < 0.0001. The three horizontal lines in the middle correspond to the first quartile, median, and third quartile in the violin plot.

This study employed a closed-loop stimulation paradigm (Fig. 5a) to verify the acceleration of spatial learning through precise modulation of place cells. The experiment was divided into three phases: first, mice freely explored the task maze for approximately 10 min, with trajectory information and neuronal electrophysiological data recorded synchronously to identify place cells; then, specific locations were randomly selected, and a single 1-s microelectrode stimulation (90 Hz square wave, intensity of 36 mV or 72 mV) was applied as the mouse passed through, lasting approximately 10 min; finally, maze testing was conducted again to assess changes in spatial cognition (see supplementary materials: Methods 1.2).

The results showed that 36 mV stimulation did not significantly alter place cell activity or spatial behavior in mice, whereas 72 mV/90 Hz stimulation induced significant changes (Fig. 5b): the spatial information content of place cells increased to 3.55 bits/spike (a 50.2% increase, Fig. 5c-i, *p* = 5.56E-4), the place field area decreased to 99.05 cm² (a 25.1% reduction, Fig. 5c-ii, *p* = 1.03E-21), and the success rate in spatial tasks increased to 51.03% (a 19.7% increase, Fig. 5d, *p* = 9.02E-3). At this intensity, both cellular activity and behavioral performance approached levels observed after 2 weeks of training, confirming its ability to accelerate the spatial learning process.

Gamma-frequency stimulation at 90 Hz may enhance Ca²⁺ influx through voltage-gated channels, potentiating NMDA receptor-dependent LTP in place cells [40]. Although 36 mV stimulation induced slight changes in behavioral success rate (Fig. S3a) and place cell firing rate fluctuations (Fig. S3b), its effect sizes (average success rate change of 6.45%, average firing rate change of −14.6%) were significantly lower than the peak responses at 72 mV (average success rate increase of 20.1%, average firing rate change of +50.2%), suggesting that 36 mV lies within a subthreshold stimulation range. From a neurophysiological perspective, the synaptic plasticity of hippocampal place cells follows Hebbian rules—long-term potentiation is induced only when the stimulation intensity is sufficient to drive postsynaptic neurons to the action potential firing threshold [41].

The selection of stimulation parameters (72 mV, 90 Hz) was based on systematic pre-experiment optimization: when testing a voltage gradient (9–90 mV, n = 5/group) at a fixed 90 Hz frequency, the 72 mV group exhibited peak changes in both Y-maze success rate and place cell firing rate (Fig. S3a-b), while the 81/90 mV groups sometimes cause animal movement interruption; when testing a frequency gradient (40–110 Hz, n = 5/group) at a fixed 72 mV voltage, the 90 Hz group again showed peak performance in both metrics (Fig. S3c). Pre-experiment data indicated that significant deviations from this parameter combination (e.g., 36 mV or 40–70/100–110 Hz) led to markedly reduced effects (Fig. S3a-d), confirming the necessity of parameter optimization. This study provides a foundational approach for closed-loop neural interface design, with potential applications for memory disorder interventions and neural repair.

## 4 Conclusion

This study successfully developed an integrated neural interface system (TS-BMA) that combines a precision-engineered bidirectional microelectrode array with closed-loop neuromodulation circuitry, enabling simultaneous high-resolution neural recording and spatially confined electrical stimulation within the hippocampal CA1 region. The system’s optimized electrode geometry and shielding design achieved localized field confinement (80 µm radius), effectively targeting place cell populations while minimizing off-target effects. Gamma-frequency (90 Hz) biphasic stimulation at 72 mV amplitude induced threshold-dependent neuroplasticity, significantly enhancing place cell spatial information content and reducing place field area, mirroring neural reorganization observed during natural learning processes. Remarkably, mice receiving 7-day TS-BMA-mediated stimulation exhibited spatial navigation performance equivalent to control groups requiring 14-day natural training, demonstrating an acceleration in spatial learning dynamics. By synchronizing stimulation with place cell activation patterns, the closed-loop platform achieved functional specificity in modulating neural population activity. These findings establish a neuro-engineering framework for precise regulation of cognitive circuits, offering transformative potential for developing closed-loop cognitive enhancement technologies, with direct applications in treating memory disorders and optimizing brain-machine interfaces.

## For studies with human subjects and animals

All animal experiments involved were approved by the Animal Care and Use Committee at the Aerospace Information Research Institute, Chinese Academy of Science.

## CRediT authorship contribution statement

**Fan Mo:** Writing – review & editing, Writing – original draft, Visualization, Methodology, Investigation, Formal analysis, Data curation, Conceptualization. **Yilin Song:** Formal analysis, Data curation. **Guihua Xiao:** Writing – review & editing, Conceptualization. **Zhaojie Xu:** Methodology, Data curation. **Shiya Lv:** Methodology, Investigation. **Wei Xu:** Visualization, Validation. **Yaoyao Liu:** Software, Resources. **Juntao Liu:** Writing – review & editing, Validation, Software. **Mixia Wang:** Methodology, Investigation. **Yirong Wu:** Writing – review & editing, Conceptualization. **Qionghai Dai:** Writing – review & editing, Conceptualization. **Xinxia Cai:** Writing – review & editing, Funding acquisition.

## Declaration of competing interest

The authors declare that they have no conflicts of interest in this work.

## Appendix Supplementary materials

Image, application 1
