# Supplementary material for: Modulation of place cells using targeted stimulation with bidirectional microelectrode arrays enhances spatial learning speed in mice
Source: Fundam Res. 2026 Jan 9;6(3):2002–10. doi: 10.1016/j.fmre.2026.01.004 (PMC13247480; doi:10.1016/j.fmre.2026.01.004)
Supplement: Supplementary file 1 [file mmc1.docx]

# Modulation of place cells using targeted stimulation with bidirectional microelectrode arrays enhances spatial learning speed in mice

Fan Mo^a,b^, Yilin Song^a,b^, Guihua Xiao^c^, Zhaojie Xu^a,b^, Shiya Lv^a,b^, Wei Xu^a,b^, Yaoyao Liu^a,b^, Juntao Liu^a,b^, Mixia Wang^a,b^, Yirong Wu^a,b^,*, Qionghai Dai^c^,*, and Xinxia Cai ^a,b^,*

*^a^ State Key Laboratory of Transducer Technology, Aerospace Information Research Institute, Chinese Academy of Sciences, Beijing 100190, China*

*^b^ School of Electronic, Electrical and Communication Engineering, University of Chinese Academy of Sciences, Beijing 100049, China.*

*^c^ Beijing National Research Center for Information Science and Technology, Tsinghua University, Beijing 100084, China.*

** Corresponding author: wyr @mail.ie.ac.cn (Yirong Wu), qhdai@tsinghua.edu.cn (Qionghai Dai) and xxcai @mail.ie.ac.cn (Xinxia Cai)*

### Materials and methods/experiment

- 1. *Surgical procedures*

To ensure a sterile environment, the operating table and all surgical instruments were thoroughly cleaned and disinfected beforehand. The mice were then anesthetized using isoflurane, with a 3% concentration for induction and 1% for maintenance. An anesthesia machine (RWD520, RWD Life Sciences, Shenzhen, China) was used for this purpose. Once anesthetized, the mice were carefully positioned and secured in a stereotaxic frame.

The left hippocampus of the mice, specifically the CA1 region (-2.54 mm anterior-posterior from bregma, -1.5 mm medial-lateral from the midline, -1.1 mm dorsal-ventral from the dura surface), was targeted for microelectrode implantation. The microelectrode was affixed to our micro-driver, allowing for vertical movement post-implantation. During the procedure, the microelectrode was precisely positioned above the intended brain region.

To ensure stability, both the micro-driver and protective sleeve were secured to the skull using dental cement. Additionally, a cranial nail was inserted over the cerebellum, serving as a ground and reference for signal recording. This configuration helped minimize signal interference from external electrical noise. The copper wire connected to the counter microelectrode was extended to connect with the cranial nail, further reducing the impact of environmental electrical noise on the recorded signals. Following the surgery, the mice were allowed a recovery period of 7 days to recuperate from the procedure.

During the surgery, the implantation speed of the microelectrode was set at 10 μm/s to ensure a careful and controlled insertion. Throughout the experimental procedure, with the mouse's head on the implantation device, the microelectrode was advanced at a speed of approximately 50 μm/s. This slow pace allowed the brain tissue sufficient time to adapt to the presence of the microelectrode.

To ensure optimal signal recording conditions and allow for recovery, the microelectrode position was maintained after adjusting the implantation depth for more than 20 minutes. This period allowed the mouse's brain tissue to stabilize and recover before the commencement of signal recording, ensuring accurate and reliable data collection.

#### Behavior Procedures

We constructed a behavioral maze to assess mice's spatial cognition objectively. The maze measures 50 centimeters in length, 40 centimeters in width, and has a path width of 10 centimeters. Mice were provided with color and shape reminder cards at each intersection to facilitate their navigation (Fig. 4a).

Before the surgery, the maze was introduced to familiarize them with the environment. The mice underwent a 2-week exploratory period in the new environment to alleviate anxiety and fear. Following this, microelectrode implantation surgery was performed, and the mice were given one week to recover.

During the light exposure period, the mice were partially food-deprived, and then randomly explored the environment during the dark period, after a one-week recovery period following surgery. During the exploration phase, food was randomly dispersed within the maze to enable the mice to explore the environment for over 30 minutes.

In the subsequent maze task, we replaced the food dispersal method by installing feeders in two corners of the maze.

Among the three different paths in the maze, we designate one as the reward path. When a mouse reaches the reward path and subsequently the feeder, it triggers the release of food rewards. If the mouse does not traverse the reward path to access the feeder, the food rewards will not be dispensed. Here, the mice explore the task maze without food rewards, and they receive food at the end of the central pathway each time they complete the full intermediate path, with each mouse undergoing 2 hours of training.

Afterward, we started the formal experiment in which we recorded both the trajectory information of the mice and the electrophysiological information of the neurons without providing any food rewards.

There was a camera in the maze or open field, with a light-emitting diode (LED) fixed onto the mouse's head. This camera captured high-resolution video at a frame rate of 30 Hz. EthoVision XT 16 was used for video recording and path extraction.

In brief, behavioral training and electrophysiological recording of place cell information are divided into three main stages: The first stage is the training process of the mice, where food-restricted mice explore a behavioral maze to find randomly scattered food. This induces the mice to run continuously, and the training lasts for 2 weeks to ensure that the mice can run continuously for over 30 minutes in the task maze. The second stage involves the recognition and memory process of the spatial environment by the mice. Here, the mice explore the task maze without food rewards, and they receive food at the end of the central pathway each time they complete the full intermediate path, with each mouse undergoing 2 hours of training. The third stage is the recording of electrophysiological signals from place cells. The mice are placed in the task maze, and their behavioral trajectory information and electrophysiological signals from place cells are recorded for about 10 minutes. During this period, no food rewards are provided. We quantified this behavioral tendency by calculating the success rate of subjects choosing the green path, which was defined as the number of times the green path was chosen divided by the total number of choices made in each trial.

The behavioral protocol for electrical stimulation consists of three consecutive phases. First, mice freely explore the task maze for approximately 10 minutes, during which their movement trajectories and neuronal electrophysiological activity in the target brain region are synchronously recorded, along with the identification of place cell channels exhibiting location-specific firing. Next, the stimulation phase begins: the experimenter randomly selects specific locations in the maze as target areas (randomly chosen for each experiment, Fig. S4a). When the mouse enters these areas, a behavioral tracking system (sampling frequency of 30 Hz) detects the subject’s position in real time and automatically triggers a single microelectrode stimulation (parameters: 40–110 Hz square wave, 1-second duration, intensity of 9–72 mV). This stimulation phase lasts approximately 10 minutes. “Random” refers to alternating different sub-locations within the preset target area for stimulation to avoid repeated stimulation at the same physical point, rather than implying randomness in the timing or conditions of stimulation. Finally, after the stimulation phase, the mice undergo the same maze test again to assess changes in their spatial cognitive abilities. The change rate in task success rate is calculated as: (post-stimulation success rate − pre-stimulation success rate) / pre-stimulation success rate. The change rate in the average firing rate of place cells is calculated as: (post-stimulation average firing rate − pre-stimulation average firing rate) / post-stimulation average firing rate.

To deeply analyze the causal chain between electrical stimulation, place cells, and behavior, the analysis strategy was conducted in a layered approach: 1) During the stimulation phase, the mouse’s behavioral trajectories and raw neuronal electrophysiological signals were synchronously recorded in real time; 2) Post-experiment, offline analysis of key place cell parameters (e.g., spatial information content, place field area) was performed, comparing changes in these parameters before and after stimulation (Fig. 5d) to elucidate the potential neural mechanisms underlying enhanced spatial cognition; 3) Micro-level place cell reorganization features (e.g., place field stability or remapping) were correlated with macro-level improvements in navigation behavior metrics (e.g., path efficiency, time to reach the target) to confirm the spatial cognition enhancement effect induced by microelectrical stimulation and its neural basis.

In the experiment, a random rotation stimulation site strategy (Fig. S4a) was employed to rule out location-specific interference. Target sub-locations were selected using a MATLAB-generated pseudorandom sequence, ensuring no spatial clustering. By randomly selecting different place fields for stimulation during the mouse’s exploration, the results reflect the overall regulatory effect of stimulation on the place cell network rather than local position dependency. Behavioral results confirmed the rationality of this design: regardless of whether stimulation was applied in the central or peripheral regions of the maze, spatial cognition in mice showed consistent improvement (Fig. S4b, p=0.379). Each of the 17 groups contained 3 biological replicates (total N=51). Although the sample size per group is small, the Kruskal-Wallis test is robust for multi-group comparisons under this experimental design.

#### Electrophysiological recording and stimulation

Starting from the first day after the surgery, the microelectrode gradually moved towards the pyramidal cell layer of the mice until it successfully reached the desired target. To record electrophysiological signals, the microelectrode utilized a combination of 4 TLC2274 and 2 TLC2272 integrated circuits, compactly packaged by Texas Instruments.

In our laboratory, we developed specialized software for spike classification and clustering. This software employed various waveform parameters, such as peak amplitude and principal components, to separate and distinguish individual neurons. Clusters containing similar waveform patterns were manually defined, ensuring the accurate identification of single units. Importantly, single units were confirmed not to exhibit a refractory period shorter than 1 ms.

The sampling rate for spike recordings was set at 30 kHz, capturing the precise timing of neuronal activity. The spike signals had a duration of 3 ms and were filtered with a cutoff frequency of 200 Hz. A threshold value of -40 μV was applied to identify significant spikes.

To stimulate the hippocampal CA1 region, an electrical stimulator, specifically the STG4002 from Multichannel Systems powered by Harvard Bioscience, Inc., was employed. MC_Stimulus II 3.5 was used to design the stimulation waveform and control the STG4002.

Regarding the latency of the closed-loop system, the video tracking system operates at 30 frames per second (fps), resulting in a frame interval cycle of 33.33 milliseconds. Electrophysiological recording involves two processes: first, neuronal signals acquired by the microelectrode array (MEA) are immediately processed through a head-mounted preamplifier for differential amplification and impedance conversion relative to a reference signal, with the preamplified signal transmitted to the recording device via lightweight cabling, showing no measurable delay throughout. In the second stage, the signal undergoes dual-channel bandpass filtering (spike signals: 250 Hz high-pass; local field potentials: 250 Hz low-pass) and is then digitized at a 30 kHz sampling rate. This results in an electrophysiological data resolution of 33.33 microseconds—three orders of magnitude finer than the video frame rate. Consequently, the maximum synchronization deviation between neural events and position updates is limited to 33.33 ms (video cycle) + 33.33 μs (electrophysiological resolution) ≈ 33.37 ms.

Using a typical mouse foraging speed of 70 cm/s as a reference, the maximum positional uncertainty = 33.37 ms × 70 cm/s = 2.34 cm. This spatial error is significantly smaller than the mouse’s body length (approximately 10 cm) and the characteristic scale of the place field map (≥10 cm). A critical metric is that the mouse’s dwell time in the stimulation-triggered target area lasts approximately 1 second—30 times the synchronization latency—ensuring reliable triggering of stimulation within the target area through this temporal buffering mechanism. Regarding stimulation latency, the MC_Stimulus II 3.5 system (Multi Channel Systems) has a hardware response time of <1 ms.

Although the 33 ms latency introduced by the video exceeds the 10 ms threshold, it has no substantial impact on location-triggered stimulation based on the following two points: (1) the positional error (2.34 cm) is smaller than the body scale and the place field resolution (about 10 cm^2^); (2) the dwell time in the target area (1000 ms) far exceeds the synchronization latency.

#### The detailed fabrication process of the microelectrode

The microelectrode utilized in our study has dimensions of 6 mm in length and 27 μm in thickness. They were equipped with 16 circular recording sites, each measuring 8 μm in diameter, specifically designed for recording place cell activity. Additionally, there was a single circular site with a diameter of 50 μm that was dedicated to electrically stimulating place cells. To ensure precise and targeted stimulation, neighboring brain regions were safeguarded against unintended electrical stimulation by an open ring. This shield site was 20 μm in width, effectively limiting the spread of electrical stimulation beyond the intended target area (Fig. 1c). COMSOL Multiphysics 5.6 was used for electric field simulation.

The fabrication process of the microelectrode involves the use of three masks. The first mask was employed to sputter a patterned platinum conductive layer onto the microelectrode, incorporating the recording sites, stimulation sites, and connecting leads. The second mask was utilized to remove the insulating layer from the microelectrode sites. The third mask was used to etch the microelectrode into its desired shape.

Here is a detailed breakdown of the fabrication steps: (1) The initial step involved cleaning the Silicon-On-Insulator (SOI) using a sequence of 98% concentrated sulfuric acid, acetone, anhydrous ethanol, and deionized water (Fig. S1a.i). (2) After cleaning, the SOI underwent thermal oxidation to create 300 nm SiO2 films (Fig. S1a.ii). (3) A layer of 5214E photoresist was spin-coated onto the surface of the SOI wafer. The photoresist was then lithographed using the first mask and exposed without the mask. The excess photoresist was dissolved in a 0.6% NaOH solution. Subsequently, a 30 nm Ti seed layer and a 250 nm Pt conductive layer are sputtered onto the patterned photoresist. The excess Ti/Pt film layer was then removed using lift-off technology in acetone (Fig. S1a.iii). (4) An 800 nm SiO2 insulating layer was deposited on the conductive layer using Plasma Enhanced Chemical Vapor Deposition (Fig. S1a.iv). (5) A layer of AZ1500 photoresist was spin-coated on the SOI surface, followed by lithography using the second mask. The excess photoresist was dissolved using a 0.6% NaOH solution. The SiO2 layer was etched using trifluoromethane plasma until all sites were exposed (Fig. S1a.v). (6) The front side of the SOI wafer was spin-coated with AZ4620 photoresist, and lithography was performed using the third mask. The excess photoresist was dissolved using a 0.6% NaOH solution. Inductive ion-coupled deep etching was then conducted on the front side of the SOI, etching the Si layer surrounding the microelectrode down to the buried oxide layer, revealing the microelectrode shape (Fig. S1a.vi). (7) A layer of BN303 photoresist was spin-coated on the front side of the SOI wafer. The wafer's front side was attached to a steel disc using a melted pitch. Subsequently, the SiO2 layer on the back side of the wafer was removed with HF acid (Fig. S1a.vii). (8) The disc with the attached wafer was immersed in a 50% KOH solution at a temperature of 80℃. This etched the bottom Si of the SOI wafer until the buried oxide layer and the microelectrode shape became visible (Fig. S1a.viii). (9) In the final step, the pitch on the disc was dissolved, resulting in the release of the fabricated microelectrodes.

#### Histological verification of the microelectrode implantation location

To prepare the microelectrode for staining, the DiI dye was initially dissolved in anhydrous ethanol. The Dil solution was carefully applied to the microelectrode, allowing sufficient time for the anhydrous ethanol to evaporate. This process was repeated three times to ensure effective attachment of the Dil dye to the microelectrode.

After implanting the microelectrode into the CA1 region of the hippocampus, they were left in place for 30 minutes. Following this, the mice's brains were removed for further processing. The brains were sequentially dehydrated using 15% and 30% sucrose solutions. Subsequently, the dehydrated brains were sectioned using a frozen sectioning machine. The staining locations of interest were then observed under a microscope, allowing for detailed examination and analysis of the microelectrode placements (Fig. S1c).

#### Modification of the microelectrode

All recording sites of the microelectrode were modified with Pt nanoparticles (PtNPs). The electro-deposition method was employed for PtNPs deposition, following the outlined procedures. In the experiments, a two-electrode configuration was set up with a Pt wire serving as the auxiliary electrode and all the sites on the microelectrode functioning as the working electrodes. A 10 mL beaker was utilized and positioned in a recirculating water bath to maintain stable conditions. The synthesis of PtNPs was performed in a 1:1 mixed solution of 48 mM H_2_PtCl_6_ and 4.2 mM Pb(CH_3_COO)_2_. The deposition process was carried out at a voltage of -1.1 V for 60 seconds. Subsequently, the microelectrode was thoroughly cleaned with deionized water to remove any residual materials (Fig. 1b).

#### In vitro electrical stimulation experiment setup

In this experiment, a petri dish with a diameter of 120 mm is first prepared, and 100 mL of phosphate-buffered saline (PBS) is added to ensure sufficient medium for the study of voltage transmission and electric field distribution. Subsequently, two microelectrode arrays are placed at the contact interface of the PBS solution, with a micro-driver used to precisely control the distance between the two electrodes. The first step of the experiment is to simultaneously drive the microelectrode arrays downward until their tips are completely submerged below the liquid surface, ensuring the stability of the electrodes and the effectiveness of voltage transmission. Next, the micro-driver begins to finely adjust the distance between the two electrodes, starting from a state where they are close to each other and gradually increasing the distance to explore the effects of distance change on voltage transmission and electric field distribution. At the stimulation and shielding sites of one microelectrode, a stable 1V voltage is applied using a Gamry Reference 600 electrochemical workstation. Meanwhile, the stimulation site of the other microelectrode array is used to detect voltage values in the PBS solution. As a control in the experiment, a 1V voltage is also applied between the stimulating microelectrode and a platinum wire at the edge of the petri dish to simulate a more extensive distribution of the electric field, and the above experiment is repeated (Fig. 2c).

#### EIS experiment setup and double-layer capacitor circuit model

EIS applies a small amplitude AC voltage to the system and measures the corresponding current response, thereby obtaining the system's impedance values at different frequencies, as shown in Fig.3b. Impedance Z is a complex number and can be expressed as:

$Z=Z'+jZ''$ (1)

Here $Z'$ is the real part (the resistive component) and $Z''$ the imaginary part (the capacitive component). It is assumed that the resistive and capacitive effects at the electrode-electrolyte interface can be described by the solution resistance $R_{s}$ (representing the resistance of the electrolyte solution), the charge transfer resistance $R_{ct}$ (related to the electrochemical reaction resistance), and the double-layer capacitance $C_{dl}$ (representing the double-layer effect formed at the interface), as shown in Fig. 3c.

Based on the circuit model $R_{s}+(R_{_{ct}}//C_{dl})$, the parallel impedance can be expressed as:

$Z_{parallel}=\frac{1}{1/R_{ct}+jwC_{dl}}$ (2)

Substituting this into the impedance expression for the entire circuit:

$Z_{\mathrm{mod}el}=R_{s}+\frac{1}{1/R_{ct}+jwC_{dl}}$ (3)

Therefore, the total impedance expression can be written as：

$Z_{\mathrm{mod}el}=R_{s}+\frac{R_{ct}}{1+jwR_{ct}C_{dl}}$ (4)

In the experiment, by measuring the impedance data at different frequencies, the frequency response impedance spectrum $Z_{f}$ can be obtained. To fit the experimental data, the impedance function $Z_{\mathrm{mod}el}$ of the equivalent circuit is used to approximate the measured impedance spectrum.

The loss function is defined to evaluate the quality of the model fit as follows:

$Loss=\sum_{i} \left|\text{Z}_{\exp}(f_{i})-Z_{\mathrm{mod}el}(f_{i})\right|^{2}$ (5)

Here, $\text{Z}_{\exp}(f_{i})$ represents the impedance values measured experimentally, $\text{Z}_{\mathrm{mod}el}(f_{i})$ represents the impedance values calculated by the model, and $f_{i}$ is the frequency at each measurement point.

Finally, as shown in Fig. 3d, the nonlinear least squares fitting method—using the fminsearch algorithm—is employed to minimize the loss function[1], $R_{s}$= 52212.0515 Ω,$R_{\text{ct}}$= 8942686.4793 Ω, $C_{dl}$= 8.7827*10^-08^ F.

The process of establishing the double-layer capacitance model typically involves measuring the electrode-electrolyte interface using Electrochemical Impedance Spectroscopy (EIS) and fitting the impedance spectra with an equivalent circuit model to extract electrochemical parameters related to capacitance and resistance[2]. We conducted EIS experiments between the stimulation site and the shield site (Fig. 3b).

During neural stimulation, the choice of the stimulation waveform plays a crucial role in determining the neuron's response. Biphasic electrical pulses are considered an effective waveform because they help reduce the electrolysis effects caused by unidirectional current, thereby minimizing tissue damage[3]. Additionally, the biphasic waveform balances ion flow during the positive and negative phases of stimulation [4].

To further study the propagation of the stimulation signal in neurons, we combined the previously established double-layer capacitance model $R_{s}+(R_{_{ct}}//C_{dl})$to analyze the signal's variations in both the time and frequency domains. Additionally, we calculated the power spectrum of the output signal.

To analyze the time-domain response and frequency characteristics of the waveform signal, we need to solve the transfer function $H(s)$ of the circuit. Based on the double-layer capacitance model circuit, the expression for the parallel impedance is:

$Z_{parallel}(s)=\frac{R_{ct}}{1+sR_{ct}C_{dl}}$ (6)

Here $s$ is the Laplace transform variable. By combining the parallel impedance with the series resistance $R_{s}$, we obtain the total impedance as follows:

$Z_{total}(s)=R_{s}+\frac{R_{ct}}{1+sR_{ct}C_{dl}}$ (7)

To obtain the system's transfer function, we relate the input signal $V_{in}(s)$ and the output signal $V_{out}(s)$ through the impedance relationship of the circuit. Since the signal received by the neuron is induced by the double-layer capacitance effect, we can apply the voltage divider principle to the circuit:

$H(s)=\frac{V_{out}(s)}{V_{in}(s)}=\frac{Z_{parallel}(s)}{Z_{total}(s)}$ (8)

Substituting $Z_{parallel}(s)$ and $Z_{total}(s)$ into the transfer function expression, we get:

$H(s)=\frac{R_{ct}}{R_{s}(1+sR_{ct}C_{dl})+R_{ct}}$ (9)

After substituting specific values for $R_{s}$, $R_{ct}$ and $C_{dl}$, the transfer function $H(s)$ becomes:

$H(s)=\frac{7.159s^{2}+9.05s}{7.2s^{2}+9.05s}$ (10)

To obtain the time-domain response, we can perform the inverse Laplace transform $H(s)$ to derive the impulse response $h(t)$, as shown in Fig. 4b. The expression is：

(11)

Here $\delta(t)$ is the unit impulse function. Using this unit impulse function, we computed the stimulation signal received by the neuron:

$V_{out}(t)=V_{in}(t)*h(t)$ (12)

To examine whether stimulation affected neural network activity, we performed frequency-domain analyses. Although the stimulation consisted of 90 Hz biphasic square-wave pulses, Fourier transform analysis showed that signal energy was distributed across the entire frequency spectrum rather than concentrated at 90 Hz (Fig. S5a), consistent with the spectral properties of square-wave signals. Power spectral density analysis of local field potentials revealed modest, non-significant increases across all frequency bands following stimulation (Fig. S5b).

#### Place cell analysis

The spike data obtained from the experiments were processed by dividing them into small grids on the floor, with each grid measuring 1 cm in width. This division allowed us to generate raw maps indicating the number of spikes and their corresponding firing locations. MATLAB R2020a was used for the place cell analysis.

To obtain more refined and informative maps, a Gaussian kernel function with a variance of 1 cm was applied to process the raw spike and occupancy maps. This processing involved dividing the spike map by the occupancy map, resulting in the creation of a smooth rate map. In addition, spikes with an average velocity of less than 2cm/s within 1 second before and after the timestamps of the spikes are not included in the analysis, and each spike location is superimposed on a Gaussian distribution centered around the corresponding recorded trajectory point.

To identify the place fields, certain criteria were employed. A place field was defined as a continuous area that exceeded 50 cm² but did not exceed 400 cm² in size. Furthermore, the peak firing rate within the place field needed to be greater than twice the average firing rate observed in the maze, and it also needed to exceed 2 Hz. If there was an interval of 5 cm or more between two firing peaks in the place field heat map, with a firing rate of zero, it was considered a distinct place field.

#### Quantification of Charge Storage Capacity and Charge Injection Limit

The charge quantity of the cathodic scan was first calculated through numerical integration, using the trapezoidal method to integrate the current-voltage curve. The integration formula is:

${CSC}_{total}=\frac{1}{v\cdot A}\times\left| \int i(E)dE \right|$ (13)

Here $v$ is the scan rate, and $A$ is the effective area of the electrode after correction. PtNPs modification forms a nanoflower structure(Fig.1b), significantly increasing the effective electrode area. The true surface area after PtNPs modification is:

$A_{real}=A_{bare}\times\frac{R_{bare}}{R_{real}}$ (14)

Where $A_{bare}$ is the bare electrode surface area, $R_{bare}$ is the bare electrode impedance， and $R_{real}$ is the actual impedance. Thus, CSC=29.49 ± 7.71 .

The charge injection limit (CIL) is defined by the maximum safe charge density:

$CIL=\frac{Q_{pulse}}{A_{real}}<0.5\times CSC$ (15)

With stimulation parameters of 72 mV amplitude, 90 Hz frequency, and 0.06 ms pulse width, CIL=1.18±0.068 .

## References

[1] Lagarias JC, Reeds JA, Wright MH, Wright PE. Convergence Properties of the Nelder--Mead Simplex Method in Low Dimensions. SIAM J Optim 1998;9:112–47. https://doi.org/10.1137/S1052623496303470.

[2] Wang S, Zhang J, Gharbi O, Vivier V, Gao M, Orazem ME. Electrochemical impedance spectroscopy. Nat Rev Methods Primers 2021;1:41. https://doi.org/10.1038/s43586-021-00039-w.

[3] Krauss JK, Lipsman N, Aziz T, Boutet A, Brown P, Chang JW, et al. Technology of deep brain stimulation: current status and future directions. Nat Rev Neurol 2021;17:75–87. https://doi.org/10.1038/s41582-020-00426-z.

[4] Günter C, Delbeke J, Ortiz-Catalan M. Safety of long-term electrical peripheral nerve stimulation: review of the state of the art. J NeuroEngineering Rehabil 2019;16:13. https://doi.org/10.1186/s12984-018-0474-8.

## Figures


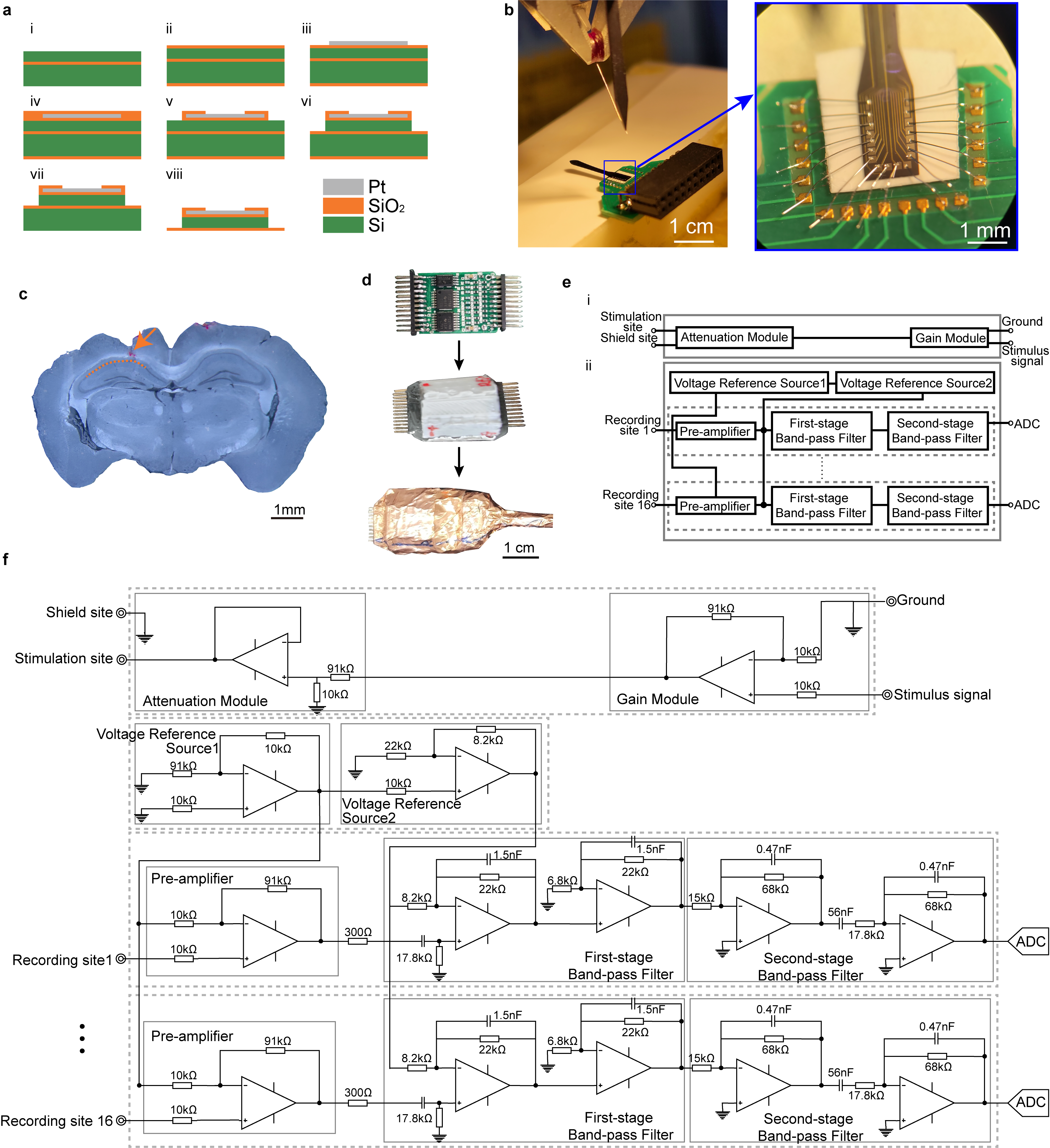


**Fig. S1. Fabrication, packaging, and bidirectional backend circuit system of TS-BMA.** (a) Process flow chart for the TS-BMA fabrication. (i) SOI substrate cleaning. (ii) Thermal oxidation. (iii) Conductive layer patterning. (iv) Insulating layer deposition. (v) Insulating layer etching. (vi) Front-side deep etching. (vii) Back-side SiO_2_ removal. (viii) Back-side Si etching. (b) Aluminum wire bonding packaging process of TS-BMA. (c) Histology showing the TS-BMA location over the CA1 cell layer in the dorsal hippocampus. TS-BMA tip stained with red Dil dye. (d) (Top) Circuit system photo, (Middle) Circuit system with plastic protection photo, (Bottom) Circuit system wrapped in aluminum foil photo. (e) Bidirectional circuit system. (i) Electrical stimulation module. (ii) Electrophysiological detection module. (f) Circuit system schematic, including: electrical stimulation module, voltage reference source, electrophysiological signal amplification and transmission path, and electrophysiological signal amplification and transmission path for Channels 1-16.


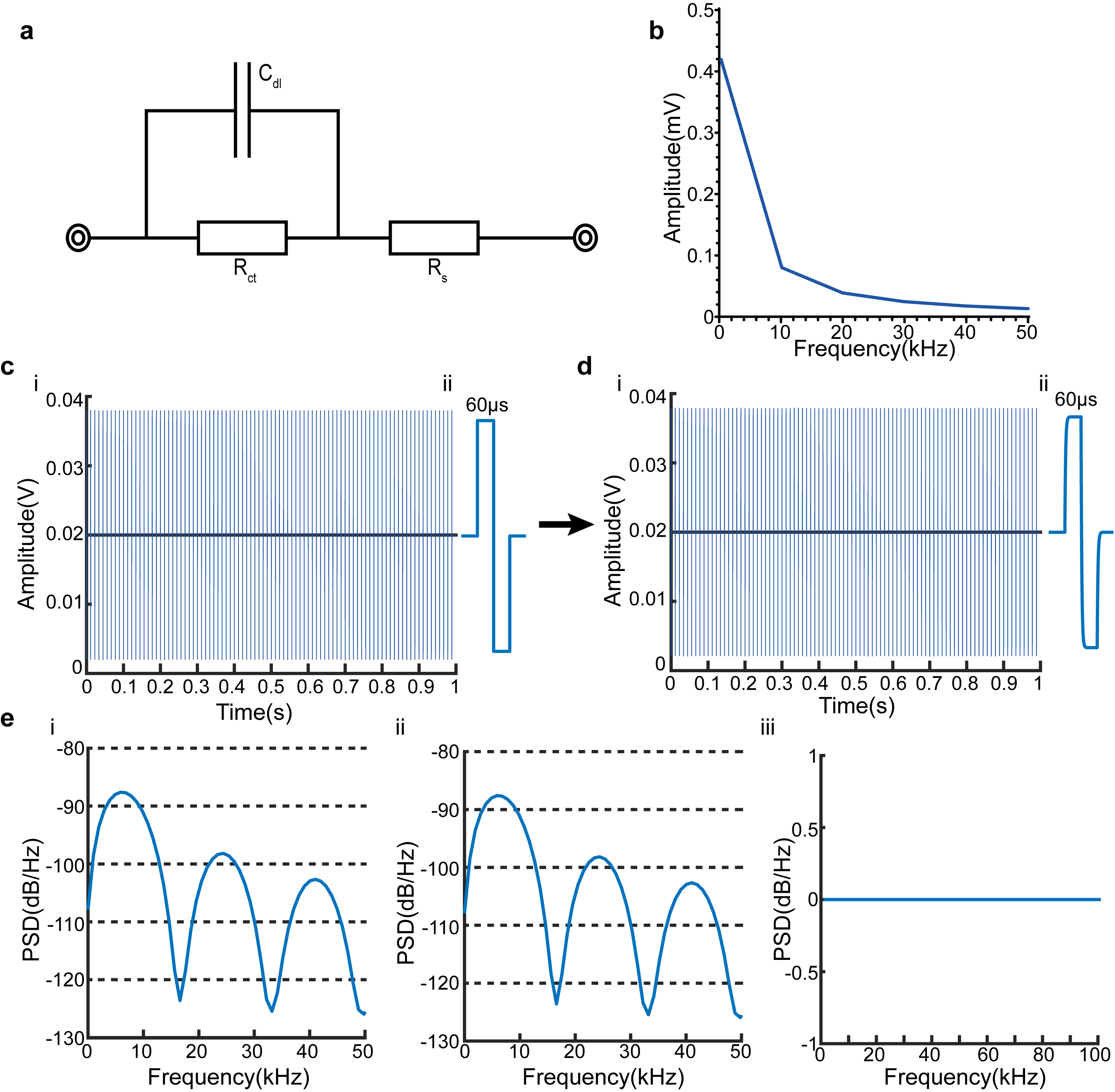


**Fig. S2. Dual capacitor model between the stimulating electrode and shield electrode.** (a) Double-layer capacitor circuit model. (b) The unit impulse response of the double-layer capacitor circuit. (c) Input waveform. (i) complete waveform (ii) single cycle. (d) Output waveform. (i) complete waveform (ii) single cycle. (e) Power spectral density. (i) Input waveform. (ii) Output waveform. (iii) Difference between input and output waveforms.


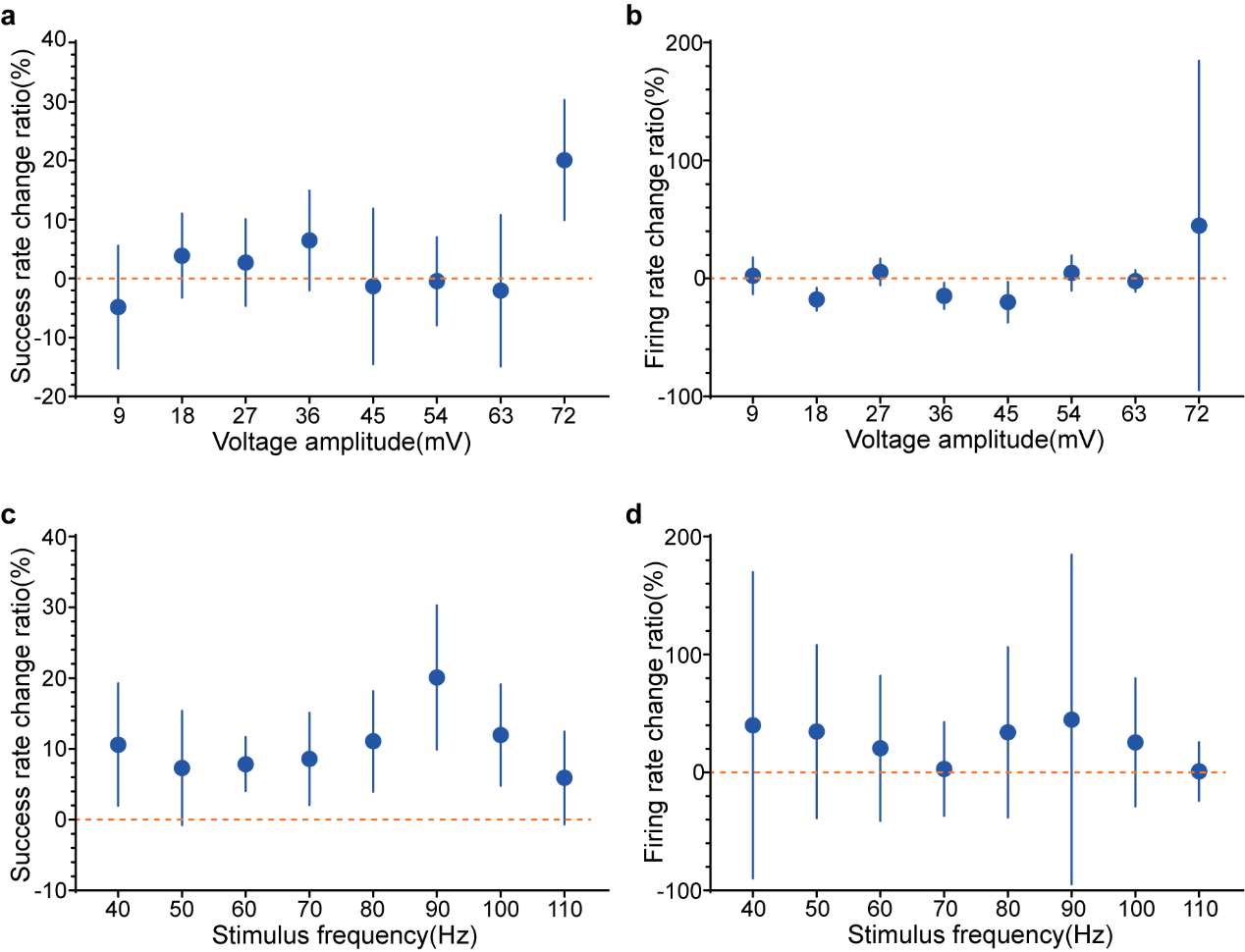


**Fig. S3 Pre-experiment results for stimulation parameter optimization (n=5 mice per group).** (a-b) With a fixed stimulation frequency of 90 Hz, the change ratios in mouse success rate (a) and place cell firing rate (b) under different stimulation voltage gradients. (c-d) With a fixed stimulation amplitude of 72 mV, the change ratios in mouse success rate (c) and place cell firing rate (d) under different stimulation frequencies. All error bars show SEM.


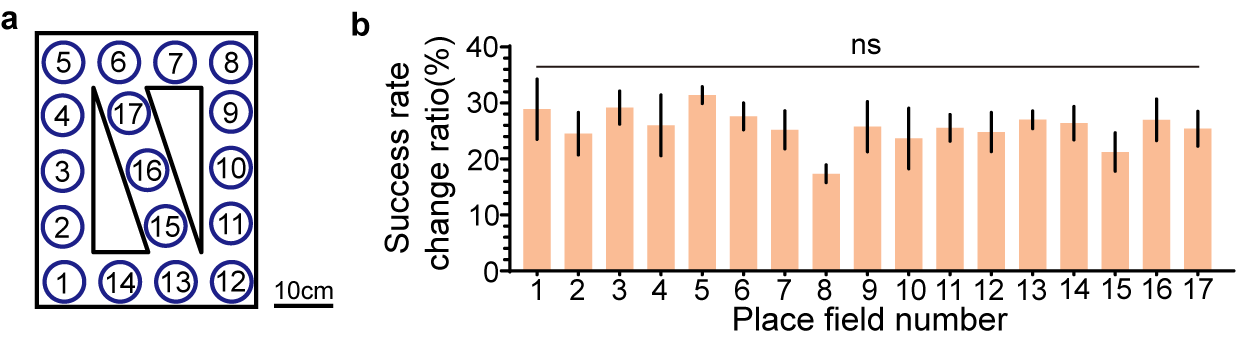


**Fig. S4 Location selection for electrical stimulation.** (a) Numbering of selectable stimulation locations. (b) Change ratios in success rate for stimulation applied at different locations (H=17.13, df=16, p=0.379, N=51, ns: not significant, Kruskal-Wallis test). All error bars show SD.


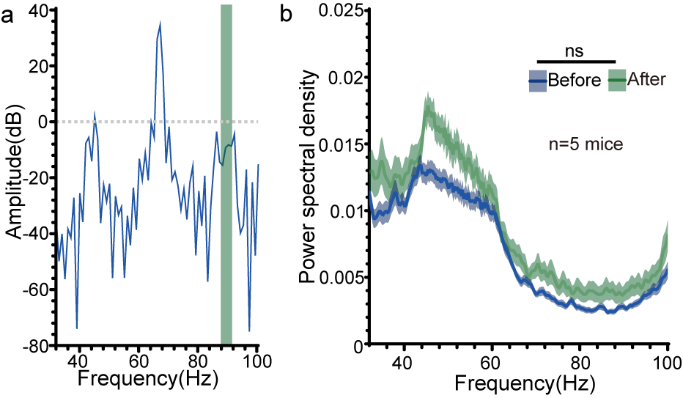


**Fig. S5. Frequency-domain analysis of stimulation signals and local field potentials.**

**(a)** Power spectrum of the 90 Hz biphasic square-wave stimulation signal. **(b)** Power spectral density of local field potentials before (blue) and after (green) stimulation. All error bars show SEM.
